# Effect of apical chloride concentration on the measurement of responses to CFTR modulation in airway epithelia cultured from nasal brushings

**DOI:** 10.14814/phy2.14603

**Published:** 2020-10-10

**Authors:** Preston E. Bratcher, Sangya Yadav, Ciaran A. Shaughnessy, Ian M. Thornell, Pamela L. Zeitlin

**Affiliations:** ^1^ Department of Pediatrics National Jewish Health Denver CO USA; ^2^ Department of Pediatrics Anschutz Medical Center University of Colorado Denver Aurora CO USA; ^3^ Dept. of Internal Medicine University of Iowa Iowa City IA USA

**Keywords:** CFTR modulator, chloride gradient, Cystic fibrosis, in vitro efficacy

## Abstract

**Introduction:**

One method for assessing the in vitro response to CFTR‐modulating compounds is by analysis of epithelial monolayers in an Ussing chamber, where the apical and basolateral surfaces are isolated and the potential difference, short‐circuit current, and transepithelial resistance can be monitored. The effect of a chloride ion gradient across airway epithelia on transepithelial chloride transport and the magnitude of CFTR modulator efficacy were examined.

**Methods:**

CFTR‐mediated changes in the potential difference and transepithelial currents of primary human nasal epithelial cell cultures were quantified in Ussing chambers with either symmetrical solutions or reduced chloride solutions in the apical chamber. CFTR activity in homozygous F508del CFTR epithelia was rescued by treatment with VX‐661, C4/C18, 4‐phenylbutyrate (4‐PBA) for 24 hr at 37°C or by incubation at 29°C for 48 hr.

**Results:**

Imposing a chloride gradient increased CFTR‐mediated and CaCC‐mediated ion transport. Treatment of F508del CFTR homozygous cells with CFTR modulating compounds increased CFTR activity, which was significantly more evident in the presence of a chloride gradient. This observation was recapitulated with temperature‐mediated F508del CFTR correction.

**Conclusions:**

Imposing a chloride gradient during Ussing chamber measurements resulted in increased CFTR‐mediated ion transport in expanded non‐CF and F508del CFTR homozygous epithelia. In F508del CFTR homozygous epithelia, the magnitude of response to CFTR modulating compounds or low temperature was greater when assayed with a chloride gradient compared to symmetrical chloride, resulting in an apparent increase in measured efficacy. Future work may direct which methodologies utilized to quantify CFTR modulator response in vitro are most appropriate for the estimation of in vivo efficacy.

## Graphical abstract

CFTR‐mediated chloride conductance in airway epithelia is commonly analyzed in the presence of a chloride gradient, which increases CFTR‐mediated changes in potential difference and transepithelial current. This study demonstrates that the presence of a chloride gradient can impact measurements of responses of CF epithelial to CFTR modulation in vitro.

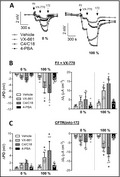

## 1 INTRODUCTION

Cystic fibrosis (CF) is an autosomal recessive disease resulting from mutations in the cystic fibrosis transmembrane conductance regulator (*CFTR*) gene that causes the encoded anion channel to be dysfunctional. While the manifestations of the disease in the airways are the major cause of morbidity and mortality in CF, improper CFTR function can give rise to symptoms in the epithelia of the upper and lower airways, sweat ducts, gastrointestinal tract, and reproductive tract. Significant improvements in lung function have been observed in patients on CFTR modulators, the most recent being elexacaftor/ivacaftor/tezacaftor (Heijerman et al., 2019; Keating et al., 2018; Middleton et al., 2019), a triple combination therapy that rescues the trafficking of the most common CF‐causing mutant, F508del CFTR, and potentiates chloride secretion. Ivacaftor, the potentiator component of elexacaftor/ivacaftor/tezacaftor, has been an effective therapy for the treatment of individuals with CF harboring gating and residual function CFTR mutations (Accurso et al., 2010; Bratcher et al., 2019; Davies et al., 2013, 2016; Guimbellot et al., 2019; Hebestreit et al., 2013; McGarry et al., 2017; McGarry & Nielson, 2013; Moss et al., 2015; Nick et al., 2020; Ramsey et al., 2011; Rosenfeld et al., 2018, 2019; Salvatore et al., 2019). Lumacaftor (VX‐809) and tezacaftor (VX‐661) are compounds that stabilize and increase expression of F508del CFTR (Molinski et al., 2018; Van Goor et al., 2011), and elexacaftor (VX‐445) functions to enhance F508del CFTR expression in the presence of VX‐661 and VX‐770 (Keating et al., 2018). Even though elexacaftor/ivacaftor/tezacaftor is effective in individuals with at least one F508del allele, thereby providing treatment for the majority of individuals with CF, there are still many CFTR mutations that are not targeted by existing CFTR modulators, including many splice site and nonsense mutations; individuals harboring two of these mutations do not have a therapy to improve the function of their CFTR. Therefore, screening for additional mutation‐specific modulators continues.

CFTR‐mediated ion transport can be measured in vivo by nasal/rectal potential difference assays and can be measured ex vivo in biopsies of the airway and gastrointestinal epitheliums (De Jonge et al., 2004; Donowitz et al., 1982; Edmonds & Godfrey, 1970; Knowles et al., 1981; Mall et al., 2000; Ussing & Zerahn, 1951; Williams et al., 1983). Electrophysiological methods to measure CFTR‐mediated ion transport in vitro include single/whole‐cell patch clamping and Ussing chamber analysis of human nasal, tracheal, bronchial, or intestinal cells (Donowitz et al., 1982; Goldfarbmuren et al., 2020; Halm et al., 1988; Kunzelmann et al., 1994; Verbeek et al., 1990; Welsh, 1986; Williams et al., 1983; Willumsen & Boucher, 1989). Each methodology possesses advantages and disadvantages.

When tissues and cultures are mounted in an Ussing chamber, the apical and basal surfaces are separated and bathed in isolated solutions (Ussing & Zerahn, 1951). Voltage and current probes are placed in each of these solutions to measure the open‐circuit potential difference or, through the use of a voltage clamp, transepithelial current. A pulse protocol allows for the measurement of transepithelial electrical conductance and its inverse, transepithelial electrical resistance. CFTR activity is evaluated either in symmetrical chloride solutions or with an imposed chloride gradient by reducing the apical chloride concentration.

Given the technical obstacles for measuring in vivo chloride concentrations, it is difficult to define the chloride gradient existing in vivo. While it is common practice to impose a chloride gradient, while maintaining ionic strength before measuring CFTR activity in an Ussing chamber, the chloride gradient commonly employed (≤2 mM chloride in the apical chamber and ~ 125 mM chloride in the basal chamber) is not physiologically relevant (Caldwell et al., 2002; Cowley et al., 2000; Grubb et al., 2002; Hull et al., 1998; Jayaraman et al., 2001a, 2001b, 2001c; Knowles et al., 1997; Kozlova et al., 2005b, 2006a, 2006b; Kozlova, et al., 2005; Matsui et al., 1998; McCray et al., 1999; Nilsson et al., 2004; Robinson et al., 1989; Song et al., 2003; Vanthanouvong et al., 2005; Vanthanouvong & Roomans, 2004). This supraphysiological chloride gradient may induce unintended effects upon the epithelium and its regulation of transcellular and paracellular ion transport. For example, imposing a chloride gradient produces voltage and current responses (Chen et al., 2010) for epithelia that likely arise from the summation of effects on paracellular chloride transport, transcellular chloride transport, and junction potentials at the probe interface. However, the amplification of the signal provided by the presence of a chloride gradient may allow for increased precision in measurements of CFTR modulator efficacy.

The testing of CFTR modulators in vitro is important for drug development and has great potential for the development of personalized medicines. Given the known impact of a chloride gradient on CFTR‐mediated ion transport, it was hypothesized that the magnitude of increase in CFTR function due to treatment with CFTR modulators may be influenced by a chloride gradient. Therefore, direct comparisons were made of measurements of CFTR function in both the presence and absence of chloride gradients in CF epithelia treated with CFTR modulators. Understanding the effects of chloride gradients of varying strengths on CFTR‐mediated ion transport may lead to better predictions of in vivo tissue‐specific responses to CFTR modulators.

## 2 METHODS

### 2.1 Collection and expansion of primary human nasal epithelia

Nasal epithelial cells were obtained by passing a nylon cytology brush (Medical Packaging Corporation) under the inferior turbinate of individuals with and without CF using a protocol approved by the National Jewish Health Institutional Review Board (HS‐2832). Prior to collection, all donors provided informed consent. Non‐CF donors included a 25‐year‐old female, a 26‐year‐old male, and a 32‐year‐old male, and donors from individuals with CF homozygous for the F508del CFTR mutation included a 25‐year‐old female, a 29‐year‐old male, and a 36‐year‐old female. Brushings were processed and expanded using previously described methods employing an irradiated NIH 3T3 feeder layer and the Y‐27632 Rho‐kinase inhibitor (ApexBIO) (Chioccioli et al., 2019; Reynolds et al., 2016).

### 2.2 Generation of differentiated airway cultures and F508del CFTR modulations

For differentiation at the air‐liquid interface (ALI), expanded cells were passaged 2–4 times and plated at a density of 250,000 cells/cm^2^ on permeable polyester Transwell inserts (Corning) coated with a 3 mg/ml of Type I bovine collagen solution (Advanced BioMatrix), grown submerged for 42–54 hr in PneumaCult ‐ Ex Plus (STEMCELL Technologies). Apical media was then removed, and basal media was replaced with PneumaCult – ALI (STEMCELL Technologies) media. Cultures were well‐differentiated and analyzed after 3–4 weeks at ALI (Chioccioli et al., 2019; Goldfarbmuren et al., 2020). Cultures are defined as well‐differentiated macroscopically by the presence of secreted mucus and microscopically by the presence of beating cilia.

For temperature correction of cells obtained from individuals with cystic fibrosis harboring homozygous F508del CFTR mutations, ALI cultures were given fresh media and transferred to 29ºC for 48 hr prior to analysis in the Ussing chamber. For chemical CFTR modulator treatments, cells were treated for 24 hr with media containing either DMSO alone (vehicle), 3 µM VX‐661 (Selleck Chemicals), 10 µM C4/5 µM C18 (CFTR Chemical Compound Distribution Program), or 1 mM 4‐PBA (Sigma).

### 2.3 Electrophysiological analysis of cultures in an Ussing chamber

Electrophysiological analyses were performed in an Ussing chamber (Physiologic Instruments) under open‐circuit conditions with intermittent voltage clamping at 0 mV for transepithelial current measurements. Cells were bathed in a modified Ringer's solution (120 mM NaCl, 10 mM D‐Glucose, 3.3 mM KH_2_PO_4_, 0.83 mM K_2_HPO_4_, 1.2 mM MgCl_2_, 1.2 mM CaCl_2_, 25 mM NaHCO_3_, pH 7.4 after saturation with 5% CO_2_/95% O_2_ gas), a gluconate‐substituted solution (115 mM NaC_6_H_11_O_7_, 10 mM D‐Glucose, 3.3 mM KH_2_PO_4_, 0.83 mM K_2_HPO_4_, 1.2 mM MgSO_4_, 5 mM CaC_6_H_11_O_7_, 25 mM NaHCO_3_, pH 7.4 after saturation with 5% CO_2_/95% O_2_ gas), or a 50:50 mixture of these two solutions. As the ionic strength was maintained in all solutions, 100%, 50%, and 0% gradients apply to both chloride concentration and activity. For reference, the calculated chloride activities were 88.48, 44.24, and 0 mM. After mounting in the chambers, solutions were continuously gassed with a 5% CO_2_/95% O_2_ gas.

**Figure 1:**
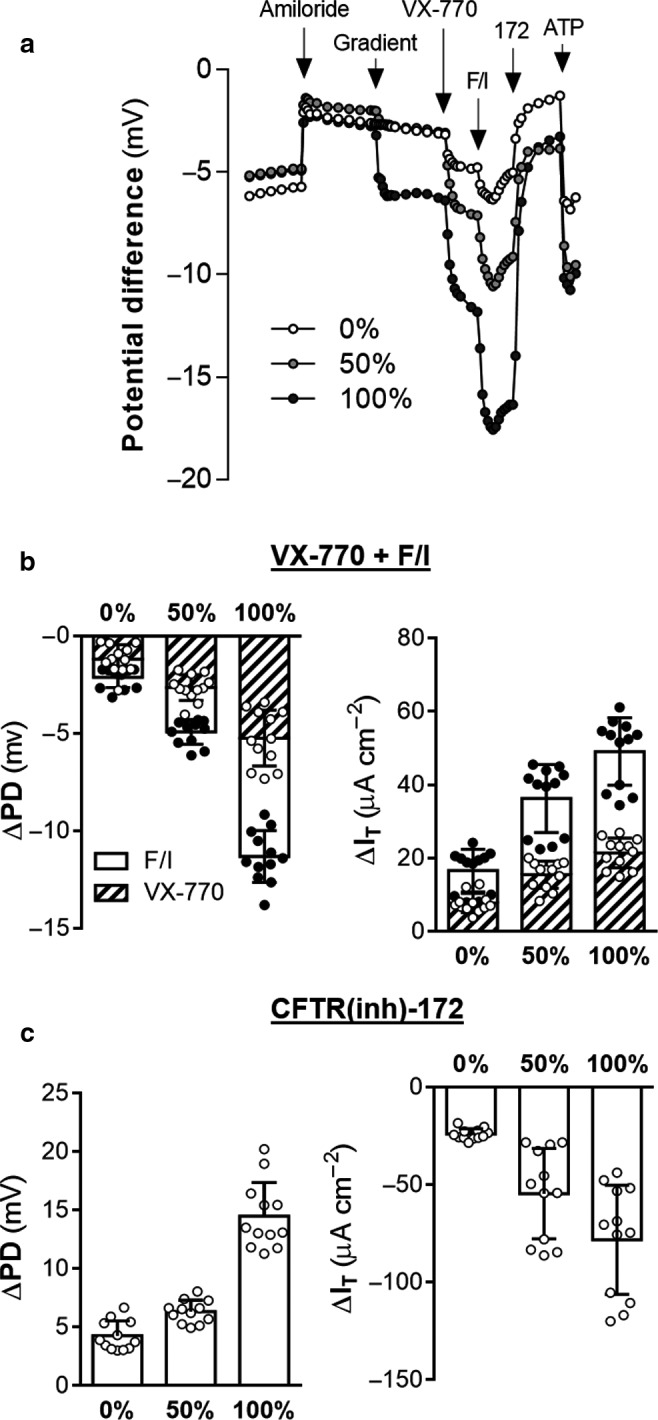
Effects of a chloride gradient on chloride transport across non‐CF nasal epithelia. Ion transport in non‐CF nasal epithelia was analyzed in an Ussing chamber under open‐circuit conditions with intermittent voltage clamping. A) Membrane potential (mV) was measured continuously in symmetrical Ringer's solution followed by amiloride (100 µM), exchange of apical buffer (containing amiloride), VX‐770 (1 µM), forskolin (20 µM)/IBMX (100 µM), CFTR(inh)‐172 (abbreviated as “172,” 10 µM), and ATP (100 µM). Changes in potential (PD) and transepithelial current (I_T_) as a result of treatments were quantified B) – C). Apical buffer was either the chloride‐containing modified Ringer's (0%), gluconate‐containing Ringer's (100%), or a 1:1 mixture of these two buffers (50%). Responses to VX‐770, F/I, and CFTR(inh)‐172 with 50% and 100% gradients were significantly increased compared to responses with symmetrical chloride (*p* < .05, Supplemental Table 1). All values are mean ± *SD* with individual data points shown; *n* = 4 per condition for each of three donors

**Figure 2:**
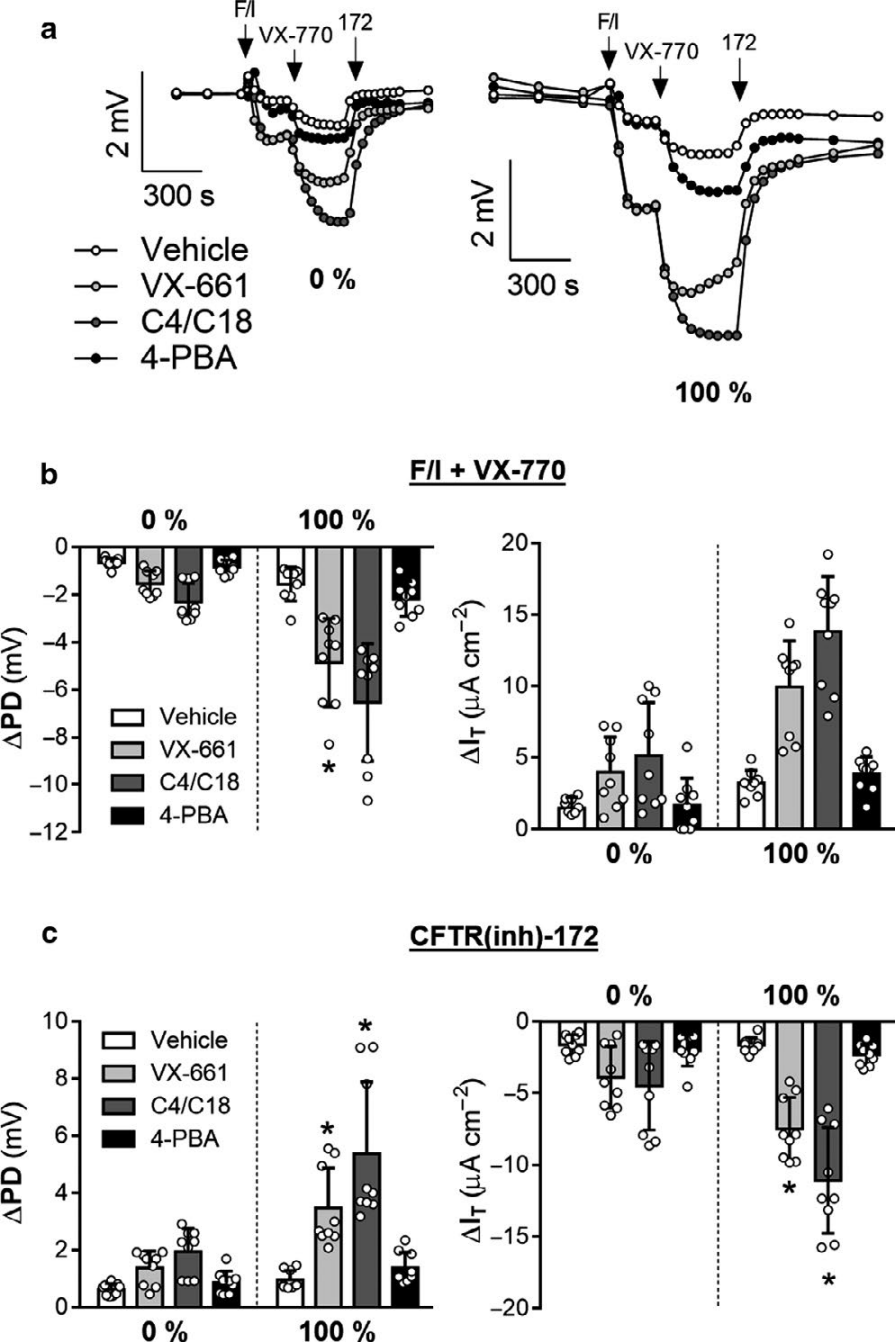
A chloride gradient increases the magnitude of response to drug correction in F508del/F508del CFTR cells. Ion transport was measured in F508del/F508del CFTR nasal epithelial cells. Cells were exposed to different CFTR modulators for 24 hr at 37ºC and then analyzed in an Ussing chamber. Representative tracings of changes in PD are in A). Changes in PD and I_T_ as a result of treatments were quantified B) – C). Asterisks denote a significant difference in the effect of a CFTR modulator at 100% (values normalized to vehicle) as compared to this effect at 0% (*p* < .05 by *t*‐test, Supplemental Table 1). All values are mean ± *SD* with individual data points shown; *n* = 3 per condition for each of three donors

Cultures were treated acutely in the Ussing chamber as depicted in Figures 1 and 2. Treatments consisted of apical 100 µM amiloride (Alfa Aesar), apical/basal 20 µM forskolin (Tocris)/100 µM IBMX (Sigma), apical 1 µM VX‐770 (Selleck Chemicals), apical 10 µM CFTR(inh)‐172 (CFTR Chemical Compound Distribution Program) and apical 100 µM ATP (Sigma). For non‐CF cultures, CFTR was potentiated with VX‐770 then the maximal CFTR response was evaluated by adding forskolin/IBMX (F/I). For CF cultures, which are nominally potentiated without F/I (Namkung et al., 2013; Van Goor et al., 2009), CFTR was first phosphorylated by treating with F/I, then VX‐770 was added.

### 2.4 Statistical analyses

Statistical analyses were performed using GraphPad Prism (Version 6.0a for Mac OS X and Version 6.0 for Windows). The D'Agostino and Pearson omnibus normality test was utilized to determine whether or not data sets were normally distributed. When normally distributed, groups were compared by either One‐way ANOVA with Tukey's multiple comparisons test or unpaired *t*‐test. When one of the more groups was not normally distributed, groups were compared by either Kruskal–Wallis test with Dunn's multiple comparisons test or Mann–Whitney test. The specific test applied during comparisons is noted in Table S1.

Z factors (Zhang et al., 1999) were used to evaluate the utility of each assay using the following formula:

$$Z=1‐\frac{3{\text{SD}}_{+\text{control}}+3{\text{SD}}_{‐\text{control}}}{\left|{\text{mean}}_{+\text{control}}‐{\text{mean}}_{‐\text{control}}\right|}$$

where Z is the Z factor and *SD* is the standard deviation of the mean. For temperature correction assays, the positive control was 29°C preincubation and the negative control was 37°C preincubation. For drug correction assays, the positive control was C4/C18 preincubation and the negative control was vehicle preincubation. 1 > Z≥0.5 indicates an excellent assay, 0.5 > Z>0 indicates a marginal assay, Z = 0 represents a yes/no type assay, and Z < 0 is an impossible screen. Negative controls are shared among drug positive controls, which were performed on the same day. Temperature‐correction studies were performed separately and have their own day‐matched controls.

## 3 RESULTS

### 3.1 A chloride gradient increases voltage and current responses to CFTR activation and inhibition in airway epithelia derived from healthy donor nasal brushings

To explore the impact of a chloride gradient on the measurement of CFTR activity in an Ussing chamber, donor‐matched non‐CF nasal epithelia, generated from expanded primary cells, were assayed in either symmetrical chloride solutions (referred to as a “0% gradient”) or with an imposed basolateral to apical chloride gradient (50 or 100%). First, amiloride was applied apically to inhibit amiloride‐sensitive sodium channels, such as the epithelial sodium channel (ENaC). Next, chloride gradients were imposed across the epithelia (Figure 1a); gradients were imposed by replacing apical chloride with equimolar gluconate (referred to as a “100% gradient”) or replacing half the apical chloride concentration with equimolar gluconate (referred to as a “50% gradient”). Cultures were then exposed to a CFTR potentiator (VX‐770), followed by forskolin/IBMX (F/I) to induce protein kinase A‐mediated CFTR activation, followed by CFTR(inh)‐172 to inhibit CFTR. The final treatment with ATP stimulated chloride channels (CaCCs).

Non‐CF nasal epithelia derived from three unique individuals was examined with and without the imposition of a chloride gradient. VX‐770 and F/I‐induced changes in potential difference (PD) and transepithelial current (I_T_) were increased by the chloride gradient. Similar results were obtained for the CFTR(inh)‐172‐mediated decrease in PD and I_T_ (Figure 1). The magnitude of these changes was dependent on the degree of chloride gradient.

### 3.2 A chloride gradient increases CFTR‐mediated voltage and current responses for modulator‐ and temperature‐corrected homozygous F508del CFTR epithelia

**Figure 3:**
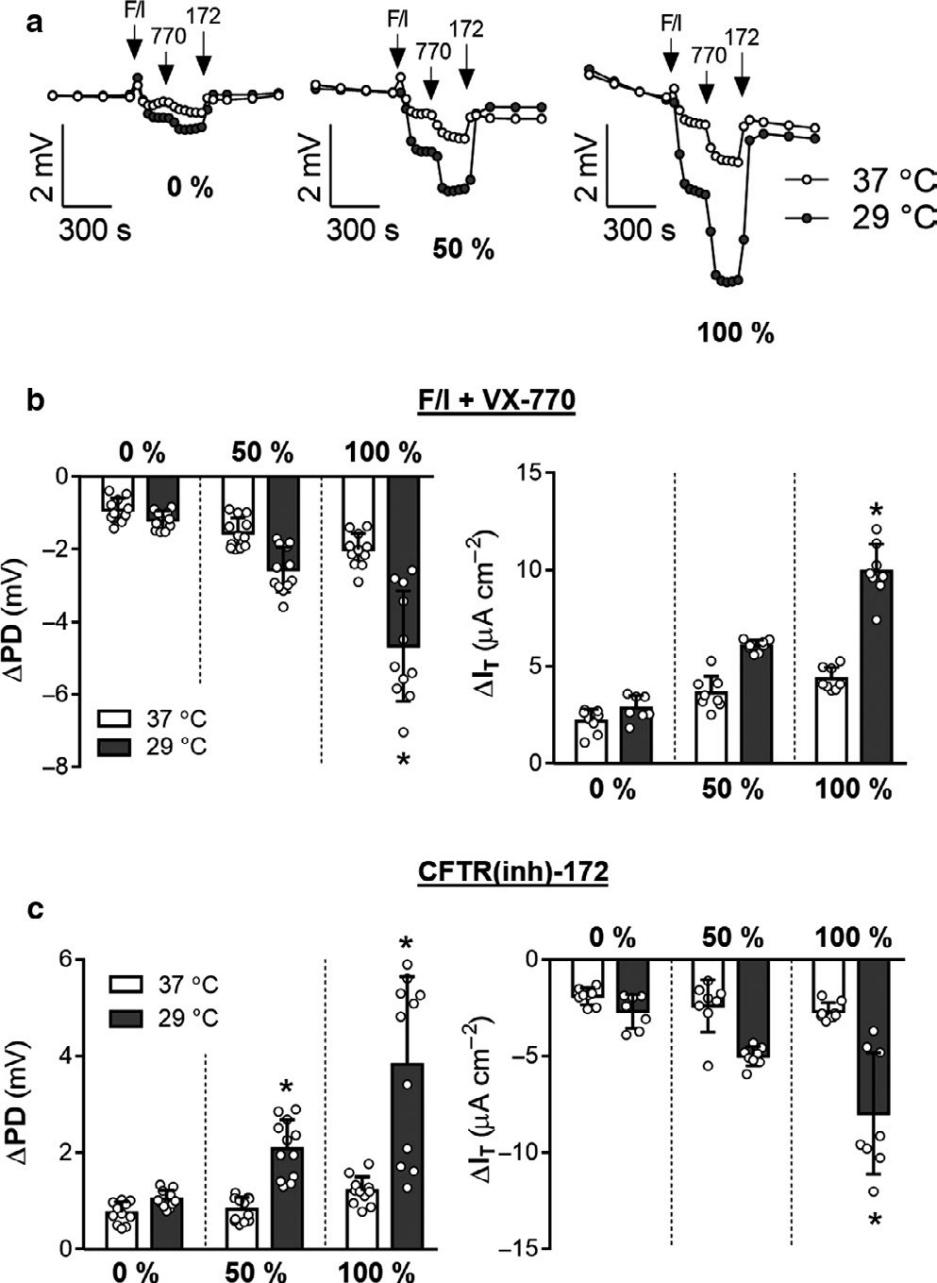
Presence of a chloride gradient increases the magnitude of the measured response to temperature correction in F508del/F508del CF cells. Ion transport was measured in F508del/F508del CFTR nasal epithelial cells in an Ussing Chamber. A) Open‐circuit data were measured in cells cultured for 48 hr at 37ºC (control condition) or 29ºC (temperature rescue of F508del). Representative tracings of cultures exposed to symmetrical Ringer's solution (left), 50% chloride gradient (middle), and 100% gradient conditions (right) are shown. Changes in PD and I_T_ as a result of treatments were quantified in B)–C). Asterisks denote a significant difference in the effect of temperature rescue (values normalized to 37º) as compared to this effect at 0% (*p* < .05 by *t*‐test, Supplemental Table 1). All values are mean ± *SD* with individual data points shown; *n* = 3–4 per condition, for each of three donors for PD and two donors for I_T_ data

Similar to non‐CF observations, CFTR‐mediated changes in PD and I_T_ elicited by post‐amiloride exposure to F/I, VX‐770, and CFTR(inh)‐172 were increased in the presence of a chloride gradient for epithelia from CF individuals with the genotype F508del/F508del (Figure 2a–c). Additionally, CFTR‐mediated changes increased in cells treated with CFTR modulating compounds that raise the expression of F508del CFTR. Donor‐derived epithelia had variable responses to modulators (Figure S1), with exposure to C4/C18 (Lopes‐Pacheco et al., 2015; Okiyoneda et al., 2013) resulting in the greatest degree of improvement. Importantly, the presence of a gradient increased the magnitude of CFTR‐mediated changes in the modulator corrected epithelia over the uncorrected epithelia. For example, VX‐661 treatment resulted in a 2.3‐fold increase in F/I/VX‐770‐mediated changes in PD when assayed under symmetrical conditions and a 3.2‐fold increase when assayed in the presence of a 100% chloride gradient (*p* = .0262, *t*‐test). This observation was reproduced for epithelia incubated at 29°C for 48 hr prior to analysis in the Ussing chamber (Figure 3a–c). As in the cultures treated with CFTR modulators, the presence of a chloride gradient increased the magnitude of the temperature‐correction effect on F508del/F508del CFTR epithelia; F/I/VX‐770‐mediated changes in PD were increased 1.4‐fold by temperature correction when assayed under symmetrical conditions and 2.4‐fold when assayed in the presence of a 100% chloride gradient (*p* = .0038, *t*‐test). It is also of note that the gradient‐induced changes in modulator response were not completely equal for the drugs tested, with C4/C18‐treated cultures having significantly greater PD responses to F/I/VX‐770 than VX‐661‐treated cultures when assayed with 0% gradient (*p* = .0271 by unpaired T‐test) but not when assayed with 100% gradient (*p* = .1241).

**Figure 4:**
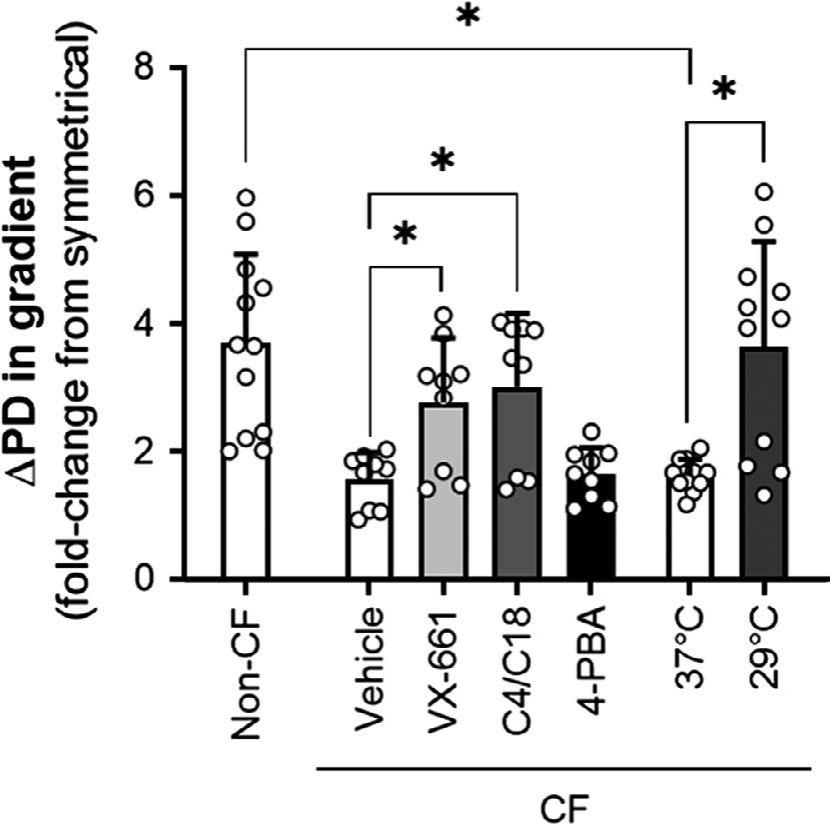
Impact of a chloride gradient on the CFTR(inh)‐172 response is greater in non‐CF and corrected CF epithelia compared to uncorrected CF epithelia. Changes in PD upon CFTR(inh)‐172 treatment in the presence of a 100% gradient were normalized by dividing with values of changes in symmetrical chloride in donor‐matched epithelia. Asterisks denote a significant difference between groups

When the changes in PD induced by CFTR inhibition in the presence of a 100% gradient are normalized to donor‐matched epithelia analyzed in the absence of a gradient, the fold difference between these conditions is greater in non‐CF epithelia than in F508del CFTR epithelia, even with the correction of the F508del gating defect using VX‐770 (Figure 4). However, the fold change in response is increased by a correction in F508del CFTR epithelia. As the presence of a gradient increased the changes in uncorrected CF epithelia less than in the corrected epithelia, this resulted in a higher measure of efficacy (defined as the fold change with treatment over the control as analyzed under the same conditions) during the gradient condition. If it can be assumed that a) VX‐661 and C4/C18 are specifically targeting F508del CFTR and b) chloride conductance through F508del CFTR is impacted to the same degree by a chloride gradient in corrected and uncorrected epithelia, then this report describes a novel phenomenon of increased measures of CFTR modulator efficacy in the presence of a chloride gradient.

### 3.3 A chloride gradient increases voltage response to ATP in cells from non‐CF and CF donors

**Figure 5:**
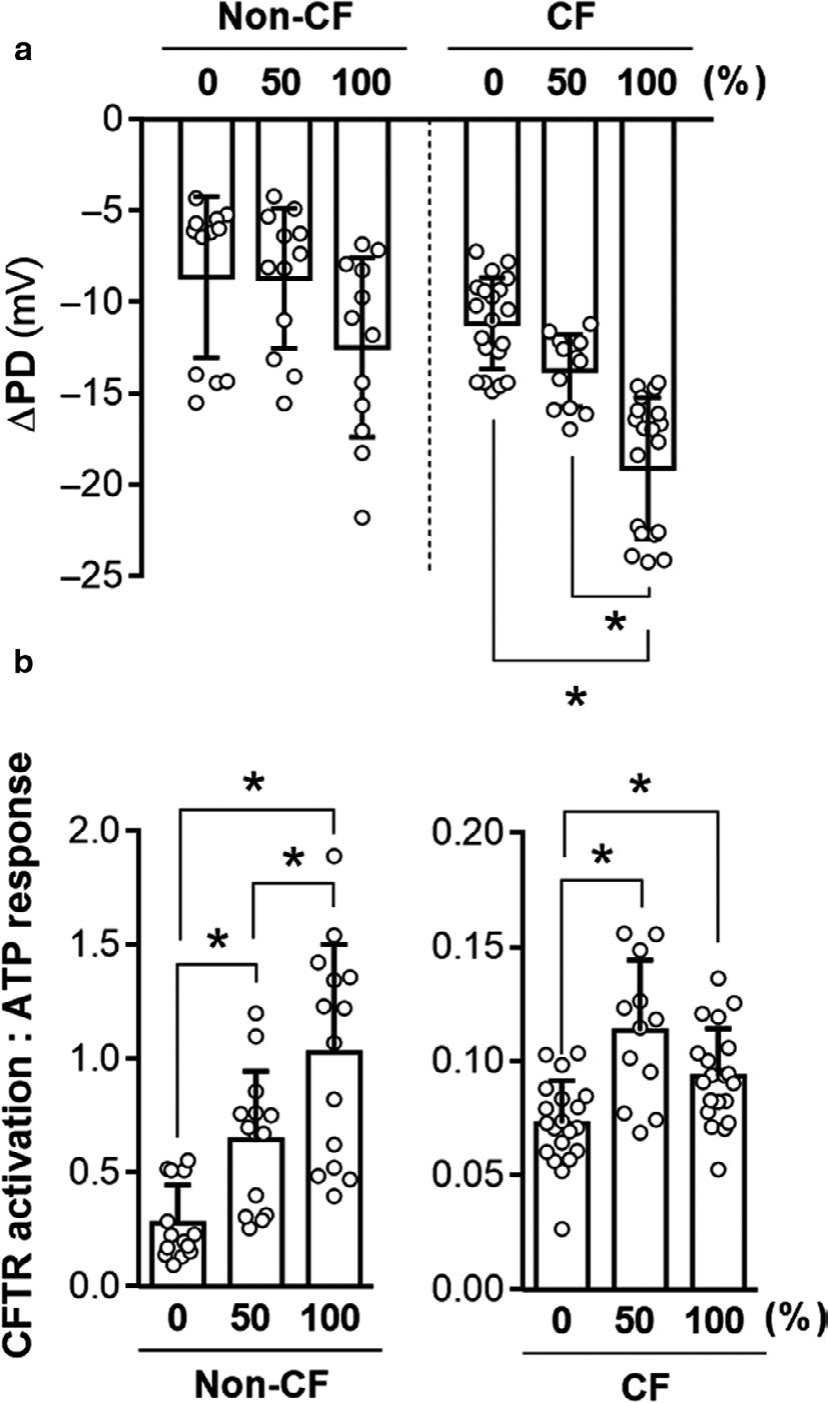
Presence of a chloride gradient increases ATP‐induced polarization in CF and non‐CF epithelia. Changes in PD after exposure of non‐CF and F508del/F508del CFTR nasal epithelial cells to ATP were analyzed in an Ussing chamber under open‐circuit conditions. A) Absolute changes in PD were quantified. B) Values of ΔPD after CFTR activation (F/I + VX‐770) were divided by values of ATP‐induced changes, resulting in a ratio of the two values. Asterisks denote significant differences between groups (*p* < .05, Supplemental Table 1). All values are mean ± *SD* with individual data points shown; *n* = 3–4 per condition for each of three non‐CF and three CF donors

ATP‐induced changes in PD were also affected by the presence of a chloride gradient in both non‐CF and CF epithelia (Figure 5a), although the donor‐to‐donor variability of this effect was substantial enough to prevent statistical significance in non‐CF cultures. However, when values were normalized to the donor‐matched 0% gradient condition values, a 100% chloride gradient significantly increased ATP‐induced responses in voltage over both 0% and 50% gradients (Figure S2a). CF cultures had significantly greater responses in PD to ATP than non‐CF cultures and, interestingly, CF culture responses to ATP were significantly impacted by both 50% and 100% gradients (Figure 5a), although significance with a 50% gradient was only achieved after donor‐matched normalization (Figure S2a, Table S1). Except for 4‐PBA (which increased the change in PD to ATP) and temperature rescue when assayed at 100% gradient, correction by CFTR modulators and low temperature had no effect on ATP responses (Figure S2a). The ratio of the response in PD during CFTR activation (F/I + VX‐770) to the ATP response demonstrates that CFTR activation is more sensitive to the chloride gradient than the ATP response, indicated by an increase in this ratio (Figure 5b). While this ratio of CFTR activity to ATP response was very modest in CF cells due to the deficiency in CFTR activity, the effect of the gradient on this was ratio was more pronounced and similar to non‐CF after correction by modulators or temperature, further supporting that changes in PD with CFTR activation are more strongly influenced by a chloride gradient than changes in ATP treatment for this protocol (Figure S2b).

## 4 DISCUSSION

The use of cultured airway epithelia is important for both CFTR modulator development and precision medicine. Therefore, it was valuable to evaluate the influence of chloride gradients on CFTR‐mediated changes in transepithelial potential difference and current in the context of non‐CF, F508del/F508del CFTR, and CFTR modulator/temperature‐corrected epithelia. Z factors were computed for temperature correction (an inexpensive method available to all laboratories culturing cells) and C4/C18 (resulted in the largest effect and, therefore, the largest signal of all tested compounds), which may both be utilized as controls for the correction of F508del CFTR expression. By Z‐factor calculations, high‐quality assay measurements were obtained for C4/C18 from analysis of CFTR(inh)‐172 responses in PD under open‐circuit conditions and in the presence of a chloride gradient (Table 1). We observed that, even for C4/C18, donor responses were variable and not always an excellent assay. The less potent correctors, VX‐661 and 4‐PBA, were less likely to yield excellent assays (Table S2). Temperature correction also resulted in lower Z‐factors in most cases, demonstrating the limited usefulness of this treatment as a control for comparison with CFTR‐modulating compounds, especially considering that only a small number of mutant CFTRs respond to this treatment. Overall, the calculations of assay quality are limited by the small sample size, and the expansion of these studies would solidify these findings. Additionally, the modulators tested may have unanticipated influences on these measurements and it, therefore, might be of importance to analyze responses to modulators over a range of assay conditions, as was performed in this study. The observation that CFTR‐mediated voltage and current changes were greater with a chloride gradient than with symmetrical solutions demonstrates the utility of increasing the driving force for chloride secretion for screening. A similar finding for CaCCs suggests that chloride gradients are useful for the screening of all chloride channels; however, why the chloride gradient was more effective for CFTR warrants further investigation. Differences in chloride secretion with a gradient also highlight that in vivo chloride gradients will affect empirical CFTR activity and apparent efficacy of CFTR modulators. The increased efficacy observed in the presence of a gradient may be due to several factors, including minimal CFTR activity in uncorrected CF epithelia or CFTR‐mediated involvement in other mechanisms of chloride transport (Castellani et al., 2012; Egan et al., 1992; Fulmer et al., 1995; Gabriel et al., 1993; LeSimple et al., 2010; Ruan et al., 2014; Sato et al., 2019). The phenomenon of increased measures of CFTR modulator efficacy was reproduced in all tested epithelia. Future research will explore the mechanism(s) involved.

**Table 1:** Evaluation of functional CFTR assay conditions using patient‐derived epithelia

Assay Conditions				Z Factor		
Gradient	+ Control	Technique	Response	Donor 51	Donor 83	Donor 150
Symmetric	29°C	Open‐Circuit	VX−770/F/I	−0.79	−3.21	−5.47
Symmetric	29°C	Open‐Circuit	CFTR(inh)−172	0.06	−1.21	−2.17
Symmetric	29°C	Voltage‐Clamp	VX−770/F/I	na	−4.76	−0.26
Symmetric	29°C	Voltage‐Clamp	CFTR(inh)−172	na	−2.44	−0.80
100%	29°C	Open‐Circuit	VX−770/F/I	0.35	0.03	−0.49
100%	29°C	Open‐Circuit	CFTR(inh)−172	0.13	0.52	−1.7
100%	29°C	Voltage‐Clamp	VX−770/F/I	na	0.24	−0.15
100%	29°C	Voltage‐Clamp	CFTR(inh)−172	na	0.30	−2.33
Symmetric	C4/C18	Open‐Circuit	VX−770/F/I	0.31	0.15	0.61
Symmetric	C4/C18	Open‐Circuit	CFTR(inh)−172	0.62	0.04	0.44
Symmetric	C4/C18	Voltage‐Clamp	VX−770/F/I	−0.76	−2.88	0.74
Symmetric	C4/C18	Voltage‐Clamp	CFTR(inh)−172	−1.46	−2.64	0.67
100%	C4/C18	Open‐Circuit	VX−770/F/I	0.31	0.39	0.67
100%	C4/C18	Open‐Circuit	CFTR(inh)−172	0.66	0.66	0.71
100%	C4/C18	Voltage‐Clamp	VX−770/F/I	0.20	0.42	0.62
100%	C4/C18	Voltage‐Clamp	CFTR(inh)−172	0.43	0.54	0.63

Note

Shaded cells represent Z factors of excellent assays. Treatment with CFTR(inh)‐172 followed treatment with VX‐770/F/I. na (not assayed). *n* ≥ 3 for donor replicates.

Regarding precision medicine, results from all the assay conditions and experimental readouts analyzed here were qualitatively similar in the evaluation of CFTR modulator responses, C4/C18 > VX‐661 > 4‐PBA, in cells obtained from F508del/F508del CFTR individuals. Nonetheless, extending in vitro modulator findings to in vivo efficacy beyond a qualitative assessment requires further study. For example, in vivo chloride gradients will depend on transepithelial driving forces and the intracellular chloride concentration, which are maintained by many transport proteins. Both of these factors will vary between individuals and by tissue‐type (e.g., nasal, tracheal, or pancreatic). Further, mimicking in vivo conditions remains a challenge, highlighted by cell culture variability among investigators using ALI cultures. Differences in the measured effects of CFTR modulators in the presence or absence of a chloride gradient introduces an important question regarding whether, and to what degree, a chloride gradient should be included for drug responses to reflect in vivo responses.

**Figure 6:**
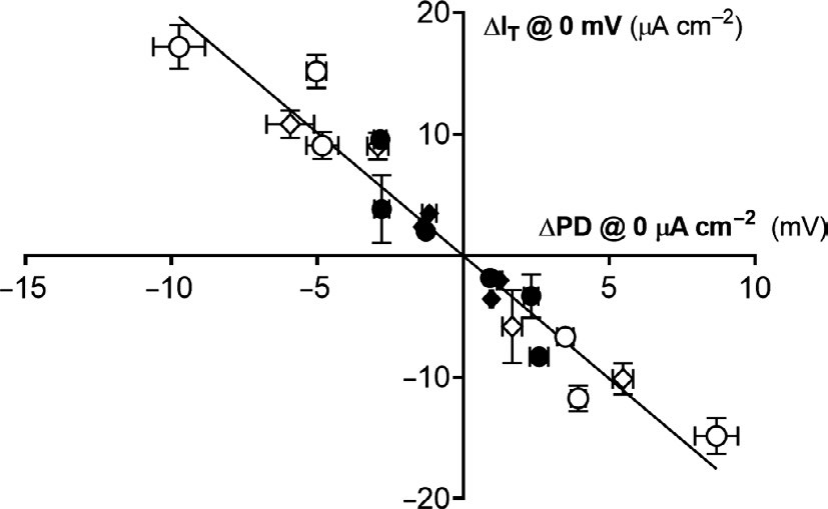
Relationship between changes in PD and I_T_ after CFTR activation and inhibition in CF epithelia. Values of ΔPD and ΔI_T_ in response to CFTR activation (F/I + VX‐770, upper left quadrant) and CFTR inhibition (CFTR(inh)‐172, lower right quadrant) (shown in Figures 2 and 3) are plotted for individual F508del/F508del CFTR donors. Diamonds represent values obtained after temperature correction at 29°C and circles represent values obtained after treatment with C4/C18. Filled symbols delineate data obtained in the presence of symmetrical chloride and open symbols indicate a 100% chloride gradient. All symbols depict mean values ± *SD*; *n* = 3–4 per condition

Direct comparisons of ΔI_T_ to ΔPD revealed an unexpected 2:1 relationship (Figure 6). While it is possible that CFTR correction and/or activation was greater under voltage‐clamping conditions as compared to open‐circuit analysis, this explanation is unlikely as both I_T_ and PD recordings were alternately performed on the same culture insert. A more likely possibility was that PD changes were limited by the influence of the paracellular pathway, whereas voltage‐clamping negated the electrical driving force of the paracellular pathway. These findings suggest that the paracellular pathway and associated electrochemical driving forces may temper transcellular flux through CFTR in vivo. The in vivo contribution* *of the paracellular pathway is difficult to quantify. Thus, nasal PD can detect in vivo CFTR rescue, but may be limited for more quantitative measurements such as evaluating in vivo potency. These technical challenges for in vivo measurements highlight the utility of in vitro preparations from patient‐derived samples. Overall, both open‐circuit PD and voltage‐clamped I_T_ measurements were effective at approximating treatment efficacies and can be used for screening potential therapeutics.

The results generated through the airway model employed in this study are subject to several caveats. It is unknown how the in vitro expansion of primary airway epithelial cells may affect their performance. However, these expanded cells formed well‐differentiated epithelia and possessed functional CFTR channels, which were absent in F508del/F508del CFTR epithelia. Native tissue would be a preferable system in which to explore the impact of a chloride gradient on ion transport; however, a voltage clamp cannot be applied across native tissue in vivo. Even preparations of intact epithelia excised from individuals or obtained from donor organs would contain mechanical damage and have the caveat that bathed solutions are controlled by the investigator. All experiments were performed using primary nasal epithelial cells as these cells generate the in vivo transepithelial electrical potential that is commonly evaluated in individuals with CF and can readily be obtained noninvasively for precision medicine studies; however, these findings may be specific for airway epithelial cells cultured from the nasal brushings. Further investigation of the gradient‐mediated effects on ion transport in native airway tissue and from tracheal and bronchial epithelial cell cultures is warranted.

These results demonstrate that a chloride gradient significantly impacts measurements of CFTR modulator efficacies in donor‐matched epithelia. In a limited number of homozygous F508del CFTR donor cultures, comparing CFTR activation versus inhibition measurements by open‐circuit PD versus voltage‐clamped I_T_ under symmetrical versus chloride gradient conditions suggest that the highest quality screening methodology may be PD measurements of inhibitor responses in the presence of a chloride gradient. Future research will determine conditions resulting in modulator responses that correlate best with in vivo responses.

## Conflict of Interest

The authors have no potential conflicts of interest for this research.

## AUTHOR CONTRIBUTIONS

P.E.B.: Conceived and designed research, Performed experiments, Analyzed data, Interpreted results of experiments, Prepared figures, Drafted manuscript, Edited and revised manuscript, Approved the final version of the manuscript. S.Y.: Performed experiments, Analyzed data, Approved the final version of the manuscript. C.A.S.: Prepared figures, Edited and revised manuscript, Approved the final version of the manuscript. I.M.T.: Analyzed data, Interpreted results of experiments, Drafted manuscript, Edited and revised manuscript, Approved the final version of the manuscript. P.L.Z.: Conceived and designed research, Interpreted results of experiments, Drafted manuscript, Edited and revised manuscript, Approved the final version of the manuscript.

## Supporting information



Supplementary Material
