# Supplementary material for: Effect of apical chloride concentration on the measurement of responses to CFTR modulation in airway epithelia cultured from nasal brushings
Source: Physiol Rep. 2020 Oct 10;8(19):e14603. doi: 10.14814/phy2.14603 (PMC7547589; doi:10.14814/phy2.14603)
Supplement: Supplementary file 1 — Supplementary Material [file PHY2-8-e14603-s001.docx]

Supplemental Tables and Figures can be located at the following link:

<https://figshare.com/s/71a41e74560020de873d>

**Supplemental Figure 1: CFTR(inh)-172-induced polarization in modulator-treated F508del/F508del CFTR epithelia.** Changes in PD after exposure of F508del/F508del CFTR nasal epithelial cells to CFTR(inh)-172 was analyzed in an Ussing chamber under open-circuit conditions. A) Data are stratified by an individual donor in order to illustrate the donor-to-donor variation in responses to modulators. B) Data normalized to vehicle-only controls demonstrate the efficacy of CFTR-modulating compounds. Asterisks denote a significant difference from the vehicle analyzed in the same condition (p<0.05, Supplemental Table 1). All values shown are mean ± SD; n = 3 per condition for each of three CF donors.

**Supplemental Figure 2: Effect of gradient and correction of CFTR expression on ATP-induced polarization in F508del/F508del CFTR epithelia.** Changes in PD after exposure of F508del/F508del CFTR nasal epithelial cells to ATP were analyzed in an Ussing chamber under open-circuit conditions. A) Changes in PD were normalized to 37°C or vehicle-alone controls. B) Values of ΔPD after CFTR activation (F/I + VX-770) were divided by values of ATP-induced changes, resulting in a ratio of the two values. Asterisks denote significant differences between groups (p<0.05, Supplemental Table 1). All values shown are mean ± SD; n = 3–4 per condition for each of the three non-CF and three CF donors.
